# Severe dengue in an adolescent with graves’ disease: a case report

**DOI:** 10.3389/fmed.2026.1990088

**Published:** 2026-10-29

**Authors:** Lin Lai, Qifeng Huang, Weilong Tan, Chongcai Wang

**Affiliations:** 1 Key Laboratory of Emergency and Trauma of Ministry of Education, The First Affiliated Hospital of Hainan Medical University, Haikou, Hainan Province, China; 2 Institute of Cancer, Hainan Academy of Medical Sciences, Hainan Medical University, Haikou, Hainan Province, China; 3 Nanjing Bioengineering (Gene) Technology Centre for Medicines, Nanjing, China; 4 Hainan International Travel Healthcare Center (Outpatient Department of Haikou Customs Port), Haikou, Hainan, China

**Keywords:** case report, dengue fever, graves' disease, hyperthyroidism, severe dengue, thyroid stimulating hormone receptor antibody

## Abstract

**Background:**

Dengue fever is a mosquito-borne viral disease with clinical manifestations ranging from mild febrile to severe disease. Mild cases may present with fever only, whereas severe cases can progress to life-threatening hemorrhage or shock. The coexistence of severe dengue and Graves’ disease in adolescents is rarely reported. Because the two conditions may share clinical manifestations, their coexistence can complicate diagnosis and management.

**Case presentation:**

We report the case of a 17-year-old female adolescent with severe dengue and preexisting Graves’ disease. The patient had an 8-year history of preexisting Graves’ disease with hyperthyroidism. In June 2025, she traveled to Yunnan Province and reported mosquito bites. In July, the patient travelled to Haikou. One week later, she developed acute nausea, vomiting, diarrhea, and persistent fever. The patient presented to a local hospital on July 15, 2025. Laboratory testing was positive for nonstructural protein 1 antigen and viral nucleic acid, confirming dengue virus infection. Serum complete blood counts showed progressive leukopenia and thrombocytopenia. Endocrinological assessment revealed a suppressed thyroid-stimulating hormone level, elevated free thyroid hormone levels, positive thyroid antibodies, and markedly elevated thyroid peroxidase antibodies (>1000 IU/mL). These findings were consistent with severe dengue, active Graves’ disease, and Hashimoto's thyroiditis. The patient received comprehensive supportive care, including continuous hemodynamic monitoring, gastric mucosal protection, antipyretic therapy, cautious fluid resuscitation, and careful management of the electrolyte and acid-base balance. Methimazole was administered to control thyrotoxicosis. Her clinical condition gradually improved, and she was discharged in stable condition with endocrinology follow-up arranged.

## Introduction

Dengue fever (DF) is a mosquito-borne acute infectious disease characterized by rapid and widespread transmission (1). Common clinical manifestations include fever, myalgia, and bleeding (2). Laboratory findings commonly include leukopenia and thrombocytopenia, and with degree of thrombocytopenia associated with increased disease severity (3). Persistent vomiting, severe abdominal pain, and altered consciousness are important clinical warning signs of progression to severe dengue (4). Laboratory indicators of potential disease progression include a rapid decline in platelet count, increased hematocrit, hypoalbuminemia (serum albumin <30 g/L), and abnormal liver function tests (4). Previous studies have identified a rapid decline in platelet count to below 50 × 10^9^/L early in the disease course as an independent risk factor for progression to severe dengue (4, 5). Furthermore, pleural or peritoneal effusion detected on imaging may indicate progression to severe disease (4).

Hyperthyroidism is an endocrine disorder characterized by excessive thyroid hormone production and a range of clinical manifestations associated with increased metabolic activity and sympathetic nervous system stimulation (6). Graves’ disease is the most common cause of hyperthyroidism, accounting for approximately 80% of cases (7). In adolescents, Graves’ disease is more common in girls than in boys, with onset occurring predominantly during early to mid-adolescence (7). Autoimmune thyroid diseases, including Graves’ disease and Hashimoto's thyroiditis, are among the most common organ-specific autoimmune diseases, and share underlying immunopathological mechanisms (8).

We report a case of severe DF in an adolescent with preexisting Graves’ disease. We describe the clinical presentation, diagnostic evaluation, and treatment of this patient to highlight the challenges associated with managing these coexisting conditions.

## Case report

The patient was a 17-year-old girl residing in Wanning City, Hainan Province. She had an 8-year history of hyperthyroidism and treated with methimazole. However, she had recently discontinued this treatment without medical advice because she believed her condition was well controlled. In June 2025, the patient traveled to Yunnan Province, where she reported mosquito bites. She had no history of international travel. On July 15, 2025, the patient presented to the emergency department with a 1-day history of nausea, vomiting, diarrhea, and fever. Her maximum reported temperature was 38.5℃, and she also experienced abdominal discomfort, mild abdominal tenderness, and fatigue. Initial laboratory testing revealed leukopenia (white blood cell count, 2.64 × 10^9^/L) and thrombocytopenia (platelet count, 53 × 10^9^/L). Considering the severity of her condition, the patient was admitted for emergency observation and further management.

On admission, her body temperature was 38.0℃, pulse rate 123 beats/min, and blood pressure 112/72 mmHg. The patient was alert and cooperative, with no remarkable facial findings. The skin and mucous membranes showed no jaundice, petechiae, or ecchymosis. Her abdomen was flat with no visible varicose veins or peristaltic waves. Further abdominal examination revealed mild tenderness and guarding, without hepatosplenomegaly. Given the unclear the cause of fever and her recent travel to Yunnan, testing for influenza A and B virus antigens, *Mycoplasma pneumoniae* antibodies, and dengue nonstructural protein 1 (NS1) antigen were ordered.

After admission, the blood routine test showed a reduction in both white blood cell (to 2.55 × 10^9^/L) and platelet count (to 48 × 10^9^/L), suggesting progressive thrombocytopenia and clinical deterioration. Electrolyte tests indicated: potassium 2.9 mmol/L, sodium 146.5 mmol/L, chloride 113.1 mmol/L. Procalcitonin was 0.17 ng/mL (reference range, 0–0.05 ng/mL), suggesting an infection. No significant abnormalities were found in the liver function, routine urine findings, fecal occult blood parameters, or coagulation function tests. Thyroid function test results were abnormal (Table 1). Laboratory results on that day for influenza A/B virus and *Mycoplasma pneumoniae* antibodies were negative, whereas the Dengue NS1 antigen was positive. According to hospital regulations, positive results were reported to the Infection Management Department, and the Longhua District Center for Disease Control and Prevention (CDC) in Haikou City was contacted for dengue virus nucleic acid testing. On July 17, the CDC reported that the patient tested positive for dengue virus nucleic acid (serotype 1) and was diagnosed with dengue virus infection.

**Table 1:** Thyroid function test results.

Parameter	Results	Reference range
Thyroid stimulating hormone (TSH)	0.23	0.35–4.94(mIU/L)
Free thyroxine (FT4)	20.23	9.01–19.05(pmol/L)
Free triiodothyronine (FT3)	6.87	2.43–6.01(pmol/L)
Total thyroxine (TT4)	162.34	62.68–150.84(nmol/L)
Total triiodothyronine (TT3)	3.34	0.54–2.96(nmol/L)
Thyroid-stimulating hormone receptor antibody (TRAb)	2.4	0–1.5(IU/L)
Thyroid peroxidase antibody (TPOAb)	>1,000	0–14(IU/mL)

To further confirm the diagnosis, the patient underwent neck ultrasonography and abdominal Computed Tomography (CT). Neck ultrasonography revealed diffuse thyroid enlargement with well-defined smooth capsular margins. The parenchymal echotexture was heterogeneous and coarse. Color Doppler flow imaging revealed markedly increased and disorganized intraglandular blood flow, consistent with the “thyroid inferno” pattern (Figure 1), which was consistent with the ultrasound characteristics of Graves’ disease. Abdominal CT revealed a fluid-density shadow in the abdominal cavity and a small amount of fluid around the gallbladder fossa, suggesting a possible serous effusion.

**Figure 1:**
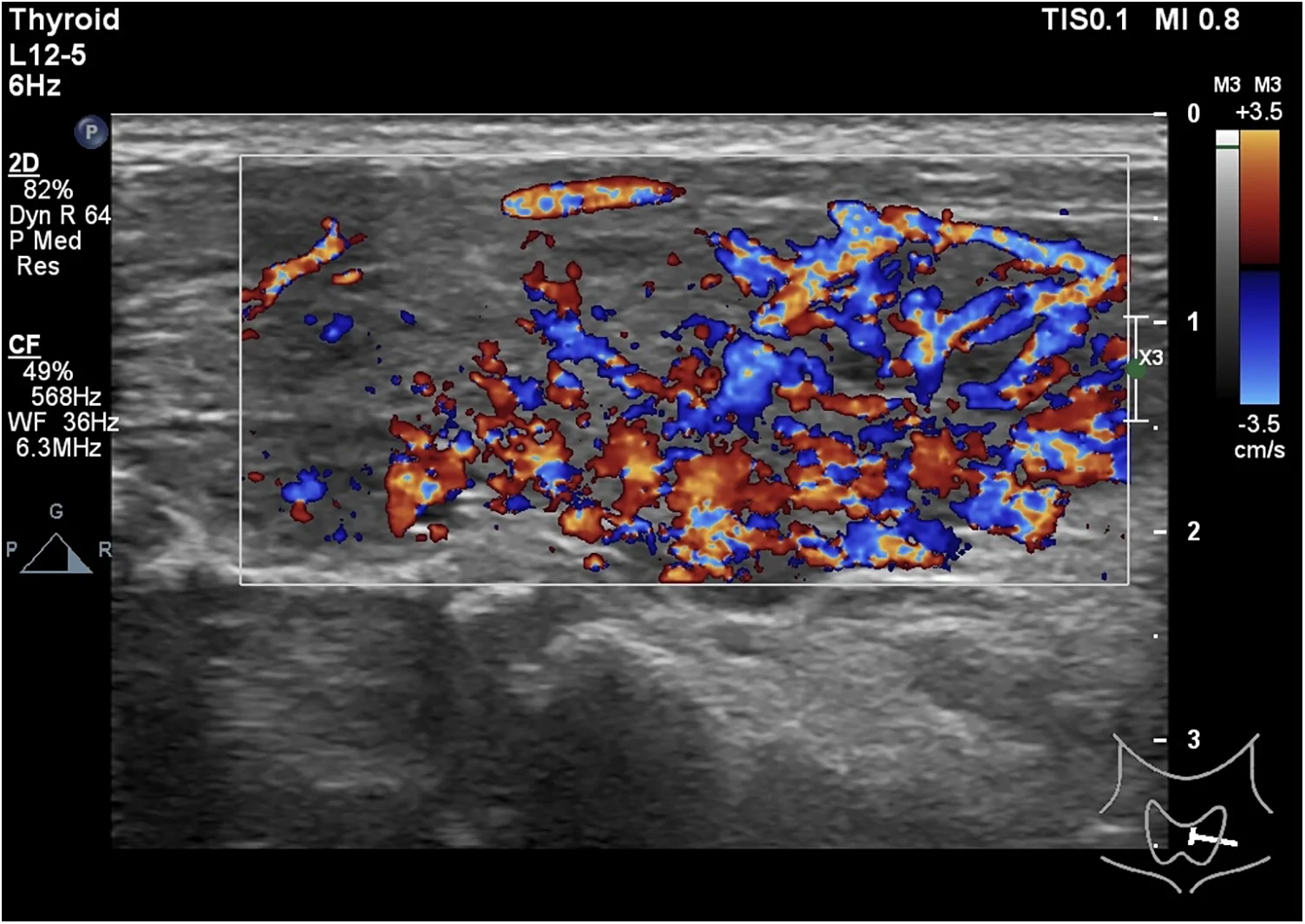
Cervical Color Doppler flow imaging.

After admission, the patient received comprehensive supportive care including gastric acid inhibition, antipyretic therapy, platelet transfusion, modulation of intestinal flora, and correction of fluid and electrolyte imbalances. To control thyrotoxicosis, the patient was started on a relatively low maintenance dose of oral methimazole along with esomeprazole to alleviate gastrointestinal symptoms and protect the gastric mucosa. After the above management, the patient’ s body temperature gradually decreased to 36.5℃ and stabilized. Fever completely subsided, and the patient's gastrointestinal symptoms were relieved. Blood routine examination showed that white blood cell count increased to 4.86 × 10^9^/L and platelet count increased to 156 × 10^9^/L, indicating improvement of the previous myelosuppression. Dynamic changes in the main indicators of routine blood tests during hospitalization are shown in Table 2.

**Table 2:** Routine blood test results.

Parameter	Day 1	Day 2	Day 4	Day 6	Day 11	Outpatient follow-up
Leukocyte count (×10^9^/L)	2.64	2.55	3.06	2.83	4.86	5.86
Platelet count (×10^9^/L)	53	48	70	66	156	174
Neutrophil(%)	24.5	11.7	23.9	23.1	83.1	51.7
Lymphocyte(%)	70.4	85.9	69.9	66.2	11.2	34.5
Monocyte(%)	3.2	1.7	3.6	7.0	4.3	7.7
Hemoglobin concentration(g/L)	47	41	75	78	92	102

On the 11th day of hospitalization, the patient's serum electrolyte levels and routine blood tests returned to normal, suggesting that the earlier multisystem dysfunction had recovered to normal levels. A repeat blood test showed that both white blood cell and platelet counts recovered to normal, reflecting effective reversal of bone marrow suppression. The patient's subjective symptoms significantly improved, and she felt well enough to be discharged. At that time, the patient was clinically stable with normal vital signs and no fever, abdominal pain, or diarrhea. The patient was advised to attend regular endocrine follow-ups to monitor thyroid function and blood counts, with dose adjustments as needed based on the results. The patient was also reminded to avoid mosquito bites and to remain alert to the possibility of dengue reinfection.

One month after discharge, a telephone follow-up revealed that the patient was clinically stable. She reported no recurrence of fever, nausea, vomiting, or diarrhea and her general condition remained good. Repeat blood counts, urinalysis, and biochemical tests revealed no significant abnormalities, indicating that the previous multisystem dysfunction had largely resolved. Thyroid function tests showed that triiodothyronine and thyroxine levels had normalized, whereas thyroid-stimulating hormone level remained below the lower limit of normal, suggesting that thyroid function was still recovering and required continued monitoring. At that time, the patient had no specific complaints and was able to resume daily activities and schoolwork without difficulty. The patient was satisfied with the treatment effects during hospitalization and provided positive feedback to the medical staff. The patient was advised to continue regular endocrinological follow-ups with methimazole dosage adjustments based on the thyroid function results. At the same time, it is necessary to pay attention to mosquito control and be alert to the possibility of DF reinfection. In general, the patient's rehabilitation process was uneventful, and short-term prognosis was favorable; however, long-term outcomes require further follow-up observation.

## Discussion

The patient presented with typical clinical symptoms of dengue, including high fever, vomiting, diarrhea, abdominal pain, and fatigue. Peripheral blood counts of white blood cells and platelets progressively decreased. Combined with the history of travel to Yunnan and the positive result of dengue virus type 1 nucleic acid confirmed by the CDC, a diagnosis of DF was established. The platelet count was reduced to 50 × 10^9^/L, and abdominal CT showed serous effusion in the peritoneal cavity and gallbladder fossa. The early warning signs of severe DF were clear. After admission, the patient received standardized symptomatic treatment, including acid suppression, gastroprotection, antipyretics, gut microbiota modulation, and fluid resuscitation, to manage electrolyte and metabolic disturbances. The patient's body temperature gradually recovered, gastrointestinal symptoms were relieved, and the peripheral blood cell count rose steadily. Subsequently, the patient entered the recovery period. Incidentally, Color Doppler flow imaging revealed markedly increased and disorganized intraglandular blood flow, consistent with the “thyroid inferno” pattern. Based on the results of thyroid function tests, a clinical diagnosis of Graves’ disease combined with Hashimoto's thyroiditis was established (6).

The pathogenic mechanisms may involve the following. Dengue virus infection triggers a strong innate immune response and induces the production of multiple proinflammatory cytokines, including tumor necrosis factor-α, interleukin-6, interleukin-1β, and interferon-*γ* (5, 9, 10). The resulting cytokine storm can exacerbate preexisting autoimmune thyroid injury by disrupting immune tolerance, activating autoreactive lymphocytes, and stimulating the secretion of autoantibodies such as thyroid antibodies (TRAb) and thyroid peroxidase antibody (TPOAb) (6). In this patient, the cytokine milieu during the acute phase likely promoted the activation of autoreactive thyroid lymphocytes, which explains the increased TRAb and TPOAb levels and clinical presentation of thyrotoxicosis (11, 12). Conversely, active hyperthyroidism may elevate the basal metabolic rate and oxidative stress burden and act synergistically with vascular endothelial damage caused by DF, thereby increasing the risk of severe complications, such as plasma leakage and organ dysfunction (6, 13, 14). Persistent immune dysregulation due to Hashimoto's thyroiditis may further inhibit the hematopoietic function of the bone marrow and increase the risk of severe cytopenia (15–17).

Dengue virus can invade the intestinal tissue and induce severe local inflammatory reactions and tissue damage (5). Pathological manifestations mainly include intestinal wall edema and shedding of mucosal epithelial cells, which cause gastrointestinal symptoms such as vomiting, diarrhea, and abdominal pain. The innate immune response stimulated by viral infections can aggravate gastrointestinal manifestations (1). A previous study reported that the incidence of vomiting in patients with DF is 29% (18).

Patients with Graves’ disease experience hypermetabolism because of the excessive thyroid hormone levels (6). High levels of thyroid hormone can interfere with the normal peristaltic rhythm of the gastrointestinal tract and delay gastric emptying, inducing or aggravating nausea and vomiting (19). In addition, thyroid hormones may directly act on the chemoreceptor trigger zone of the central nervous system, the vomiting center, and participate in the excitation of the vomiting reflex (20). Therefore, viral infection and hyperthyroidism may synergistically promote symptoms of digestive tract discomfort via different pathways.

In patients with Graves’ disease alone, thyroid hormone levels can be maintained in a relatively stable range using antithyroid medications, and the risk of a thyroid storm is low. However, when combined with dengue infection, high fever, dehydration, and extensive release of inflammatory cytokines constitute important risk factors for precipitating a thyroid storm (21). DF is one of the most common and dangerous triggers of a thyroid storm in patients with Graves’ disease (22). A thyroid storm is an acute exacerbation of hyperthyroidism that endangers patient lives. Classic manifestations include gastrointestinal symptoms such as nausea, vomiting, and diarrhea (22). Accordingly, when patients with Graves’ disease have DF, multiple mechanisms such as viral infection, thyroid dysfunction, and pre-critical symptoms may contribute to gastrointestinal discomfort (23). These overlapping symptoms significantly increases the difficulty of clinical differentiation.

In terms of hemodynamic characteristics, patients with Graves’ disease alone are in a hypermetabolic state, manifested as peripheral vascular dilation and increased cardiac output; that is, a hemodynamic pattern of high output and low resistance (6). In contrast, a core pathological change in DF involves capillary leakage, leading to plasma extravasation and a significant reduction in effective blood volume (5, 14, 24). When these two pathological states overlap, hypovolemia induced by DF may be substantially amplified by the vasodilation associated with hyperthyroidism. Therefore, these patients have a considerably higher risk of hypovolemic shock and serous cavity effusion (pleural effusion and ascites) than those with DF alone. The disease progresses rapidly, and the requirements for fluid resuscitation strategies are more complex and individualized. These hemodynamic interactions further underscore the clinical complexity of this comorbidity.

Methimazole is a first-line thioamide drug used to treat Graves’ disease; however, its adverse effects have been reported (25–27). Drug-induced neutropenia usually progresses with continuous drug administration and resolves slowly after withdrawal. This feature emphasizes the importance of a careful differential diagnosis of the viral leukopenia. In this case, methimazole was administered before the complete resolution of dengue-associated inflammation, which theoretically constitutes a clinical dilemma in which viral and drug-induced hematopoietic toxicities overlap. However, serial blood counts obtained throughout the clinical course did not reveal progressive neutropenia, and no hematological or hepatic adverse events related to methimazole were recorded. The relatively low maintenance dose and the short duration of acute DF may be the main reasons for the absence of drug-related adverse reactions.

Currently, only two live-attenuated dengue chimeric vaccines are licensed for global use. They were developed by Sanofi Pasteur and Takeda Pharmaceuticals and confer protection against dengue in susceptible populations (28). However, both vaccines have limitations with respect to the eligible populations and protective efficacy (28, 29). Furthermore, no specific antiviral drug for dengue virus exists, and treatment is mainly based on symptomatic support. Patients with severe dengue require appropriate hospital management. Treatment measures include fluid resuscitation to maintain tissue and organ perfusion; anti-shock therapy; red blood cell and platelet transfusion for severe bleeding; and strengthening the protection of the heart, liver, kidney, and other important organ functions (30, 31). Rehydration therapy is a key link for populations at high risk for severe dengue. If a patient has warning signs of severe disease or plasma leakage, early intravenous fluid administration may help reduce disease severity (31). For severe plasma extravasation, especially in hypoproteinemia, human serum albumin infusion should be promptly administered (32). A reasonable rehydration strategy can effectively prevent the occurrence or progression of shock (32). In addition, the blood lactic acid level should be dynamically monitored in patients with severe DF, and improvement in the lactic acid value is an important monitoring indicator, which is of great significance for guiding fluid resuscitation and the use of vasoactive drugs (33). In general, the diagnosis and treatment strategy for DF should follow the core principles of early identification, diagnosis, isolation, and symptomatic support (31).

This case demonstrates that even if only one viral serotype (DENV-1) is identified, severe DF can still occur in the context of a potentially unfavorable host-related immune metabolic background. In these cases, a stratified surveillance strategy is recommended. During the acute phase, complete blood counts and liver and kidney function tests should be performed daily to dynamically monitor changes in platelets, granulocytes, and transaminases to distinguish viral from drug-induced organ damage over time (4). Thyroid function and autoantibodies should be assessed regularly, and the dose of methimazole should be adjusted according to the hormone levels to avoid aggravation of vascular leakage caused by hyperthyroidism and reduce the occurrence of severe disease and drug-related complications (26, 34).

This study had an inherent limitation of being a single-case retrospective analysis. Due to the lack of dynamic comparative data on thyroid-related cytokines and inflammatory factors before and after infection, it is difficult to fully elucidate the precise molecular pathways of thyroid autoimmunity activated by the dengue virus. The short follow-up period limits our understanding of the long-term stability of thyroid function and the evolution of the autoimmune status after dengue infection. The absence of a control case limits assessment of the extent to which hyperthyroidism contributes to the clinical presentation and severity of dengue. In the future, accumulating more similar complex cases and conducting retrospective studies within a multicenter framework will help reveal the interaction mechanism and provide more reliable evidence for clinical management.

## Conclusions

This case provides clinical evidence for the combined management of severe dengue, Grave's disease, and Hashimoto's thyroiditis. These observations suggest that dengue-induced cytokine release may induce or aggravate autoimmune thyroid dysfunction in susceptible individuals. Conversely, preexisting hyperthyroidism may exacerbate dengue-associated plasma extravasation and cytopenia by interacting with immune and metabolic mechanisms. Antithyroid therapy is feasible for the treatment of acute dengue. However, clinicians should closely monitor the hematological parameters of these patients. Early recognition of the warning signs of severe dengue and timely targeted and symptomatic treatment are of great significance for reducing morbidity, promoting clinical recovery, and improving long-term health management.

## Patient perspective

The parents of the patient reported that, following symptomatic and supportive treatment including fluid resuscitation and antipyretic therapy, the child's clinical condition improved markedly, with notable alleviation of both symptom severity and overall disease severity. The family expressed a high level of satisfaction with the diagnostic and therapeutic protocol and its efficacy in the management of dengue fever.

## Data Availability

The original contributions presented in the study are included in the article/Supplementary Material, further inquiries can be directed to the corresponding authors.
